# Multimorbidity Is Associated With Pain Over 6 Years Among Community-Dwelling Mexican Americans Aged 80 and Older

**DOI:** 10.3389/fpain.2022.830308

**Published:** 2022-03-23

**Authors:** Sadaf Arefi Milani, Mukaila A. Raji, Yong-Fang Kuo, David S. Lopez, Kyriakos S. Markides, Soham Al Snih

**Affiliations:** ^1^Division of Geriatrics and Palliative Medicine, Department of Internal Medicine, University of Texas Medical Branch, Galveston, TX, United States; ^2^Sealy Center on Aging, University of Texas Medical Branch, Galveston, TX, United States; ^3^Center for Interdisciplinary Research in Women's Health, University of Texas Medical Branch, Galveston, TX, United States; ^4^Department of Preventive Medicine and Population Health, University of Texas Medical Branch, Galveston, TX, United States; ^5^Office of Biostatistics, University of Texas Medical Branch, Galveston, TX, United States; ^6^Department of Nutrition, Metabolism, and Rehabilitation Sciences, University of Texas Medical Branch, Galveston, TX, United States

**Keywords:** multimorbidity, pain, chronic disease, older adults, Mexican Americans

## Abstract

**Introduction:**

Multimorbidity, the co-occurrence of two or more chronic conditions, is common among older adults and is associated with decreased quality of life, greater disability, and increased mortality. Yet, the association of multimorbidity with pain, another significant contributor to decreased quality of life, has not been widely studied. This is especially understudied among very old (aged ≥ 80) Mexican Americans, a fast-growing segment of the United States (US) population.

**Objective:**

To assess the association of multimorbidity with pain in very old Mexican Americans, over six years of follow-up.

**Methods:**

We used data from Waves 7 (2010/2011) to 9 (2015/2016) of the Hispanic Established Populations for the Epidemiologic Study of the Elderly, a longitudinal study of older Mexican Americans residing in the Southwestern US. Multimorbidity was defined as reporting two or more chronic health conditions. Pain was defined as (1) pain on weight-bearing, (2) pain in back, hips, knees, ankles/feet, legs, entire body, or two or more locations, and (3) pain that limits daily activities. We use generalized estimation equations to estimate the odds ratio of pain as a function of multimorbidity over 6 years.

**Results:**

At baseline (*n* = 841), 77.3% of participants had multimorbidity. Those with multimorbidity had greater odds [2.27, 95% confidence interval (CI): 1.74, 2.95] of reporting pain on weight-bearing over time, compared to those without multimorbidity. Also, those with multimorbidity had 2.12 times the odds of reporting pain that limited their daily activities (95% CI: 1.61, 2.78) compared to those without multimorbidity. Lastly, those with multimorbidity had higher odds of reporting pain in their back, knee, ankles/feet, legs, hips, entire body, or two or more locations, compared to those without multimorbidity.

**Conclusions:**

Those with multimorbidity consistently had higher odds of all types of pain, highlighting the need for early management of pain among those with multiple chronic conditions and complex health needs. This is especially important among very old Mexican Americans, who have a high burden of chronic health conditions.

## Introduction

The worldwide population is rapidly aging. The proportion of the population aged 60 and older is expected to nearly double, from 12% in 2015 to 22% in 2050 (1). In the United States (US), older adults are one of the fastest growing groups and the proportion of the population aged 65 and older is expected to increase from 16% in 2019 to almost 22% by 2040 (2). Mexican Americans are one of the fastest growing ethnic groups in the US and make up a majority (>65%) of the Hispanic population (3, 4). This large shift in the age structure of the population represents a challenge, as older adults often have complex health needs.

Pain is common among adults in the US, particularly among older adults and women. In 2019, among those aged 65 and older, 30.8% had chronic pain and 11.8% had high impact pain, defined as chronic pain that limits life or work activities (5). Potentially modifiable factors associated with chronic pain include physical inactivity, sleep problems, depression, and obesity (6). Pain is a major contributor to decreased physical function (7), greater mortality (8), and decreased quality of life (9). Yet, pain is underrecognized and under treated in Black and Hispanic populations (10, 11). Findings are mixed regarding the burden of pain among Hispanic Americans. A review article found that Hispanic Americans reported fewer pain conditions but more severe pain compared to non-Hispanic White participants (12). Another study using the Health and Retirement Study (HRS) found that Hispanics and non-Hispanic Blacks reported more severe pain compared to non-Hispanic Whites, but Hispanics and non-Hispanic Whites reported less activity impairment due to pain compared to non-Hispanic Blacks (13). In adjusted analysis of adults aged 50 and older in the HRS, Hispanic and non-Hispanic Whites were similar in their odds of high-impact chronic pain (i.e., pain lasting 7 or more months that substantially impacts daily activities) (14) and of often being troubled with pain (15). Another understudied but clinically relevant area is the virtual absence of syndemic approaches and frameworks in the examination of the relationship between co-occurring multiple chronic conditions (multimorbidity) and the incidence and correlates of overlapping pain conditions (16–18).

Multimorbidity, defined by the World Health Organization (WHO) as “the coexistence of two or more chronic conditions in the same individual,” is common among older adults and women (19). A literature review of 52 studies estimated that the prevalence of multimorbidity among older adults (65 and older) in high-income countries was 66.1% (20). Pain is prevalent among older adults with multimorbidity (6, 21–25). This is concerning, as chronic pain and multimorbidity have shared risk factors, such as age, gender, frailty, and physical inactivity, as well as shared adverse outcomes, including disability, decreased quality of life, greater psychological distress, and increased mortality (22, 26). Multimorbidity has been associated with pain (21, 23–25, 27); however, few studies have characterized the association between pain and multimorbidity (27), for instance examining pain sites and impact on activities of daily living.

Mexican Americans are an important group to study because of the previously documented Hispanic Paradox (28, 29), which first documented that the health status of Hispanics was similar to that of non-Hispanic Whites, although they were more disadvantaged in terms of socioeconomic status and other health indicators (diabetes, obesity, infectious diseases). More recent work noted an advantage in mortality, particularly among older Mexican American adults (30). Although the findings on pain among Hispanics are mixed, previous work using the 2010-2017 National Health Interview Survey found that individuals of Mexican origin have lower rates of severe and chronic pain compared to White Puerto Ricans and non-Hispanic Whites (31). A systematic review of studies published from 2000 to 2020 documented that Hispanic adults, including Mexican Americans, are less likely to receive prescription analgesic medications, compared to non-Hispanic White adults (10).

Previous work examining racial/ethnic differences among middle-aged adults in the US found that, while Hispanic adults had fewer chronic diseases at baseline, they accumulated chronic diseases 1.5% faster than non-Hispanic Whites (32). Older Mexican Americans more often report chronic conditions, including diabetes, osteoarthritis, and hypertension, compared to non-Hispanic Whites (33). Yet, it is not clear whether the greater burden of chronic diseases translates to greater odds of pain, given the lower rates of pain documented among Mexican Americans (31).

The objective of this study was to assess the association of multimorbidity with pain over 6 years of follow-up. We examined pain on weight-bearing, pain that limits daily activities, and pain site. We hypothesize that multimorbidity will be associated with greater pain among Mexican Americans aged 80 and older.

## Methods and Materials

### Population

We used data from Waves 7 (2010/2011), 8 (2012/2013), and 9 (2015/2016) of the Hispanic Established Population for the Epidemiologic Study of the Elderly (H-EPESE), a longitudinal study of older Mexican Americans residing in the Southwestern US (30, 34). The H-EPESE began in 1993/1994 with adults aged 65 and older (*n* = 3,050). In 2004/05 a new cohort of 902 participants aged 75 years and older were added to the 1,167 participants of the original cohort who were 75 years and older (*N* = 2,069). Bilingual interviewers were trained to gather information on socio-demographics, health conditions, and psycho-social characteristics of respondents in their language of choice every 2 or 3 years. Detailed information on the H-EPESE is publicly available at the National Archive of Computerized Data on Aging (35).

**Figure 1:**
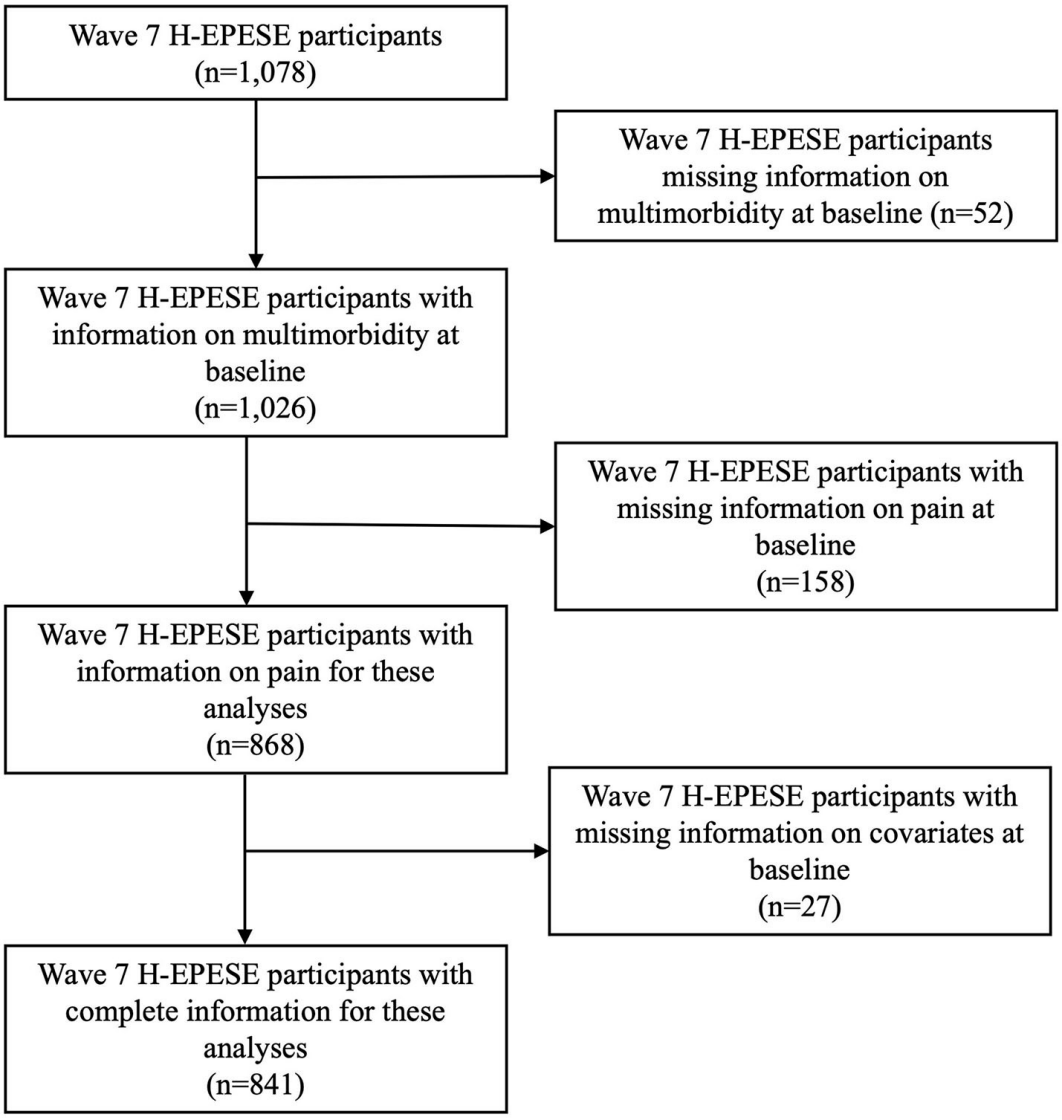
Analytic sample selection. H-EPESE, Hispanic Established Populations for the Epidemiologic Study of the Elderly.

Wave 7 was used as the baseline for these analyses, where participants were aged 80 and older (Figure 1). Of the 1,078 participants interviewed at baseline, we excluded participants who had missing information on health conditions included in our multimorbidity variable (*n* = 52), those with missing information on pain at baseline (*n* = 158), and those with missing information on covariates (*n* = 27). Our final analytical sample was 841. Those excluded from our analysis had fewer years of education on average, lower Mini-Mental State Examination Score (MMSE) scores, and more often reported high depressive symptoms, pain on weight-bearing, and pain that limited their daily activities (Supplementary Table 1). Although the H-EPESE allows for direct or proxy interviews, information on pain and the MMSE were obtained by direct interviews with the participant. Therefore, our final analytic sample did not include proxy interviews.

### Measures

#### Independent Variable

Multimorbidity was defined as two or more chronic conditions, based on the WHO's definition (19). The conditions included in this definition were diabetes, hypertension, arthritis, heart attack, heart failure, hip fracture, osteoporosis, liver disease, kidney disease, and chronic obstructive pulmonary disease. Participants were asked if they have ever been told by a doctor if they had each condition at each wave. Once participants reported a condition, they were categorized as having that condition at future waves.

#### Dependent Variable

Participants were asked if they experienced pain in the previous month when walking or standing (pain on weight-bearing). Those who respond yes were then asked, “where was this pain” (back, hips, knees, ankles/feet, legs, entire body, somewhere else) and “does this bodily pain last for four or more weeks?” (yes/no). We created a pain site score variable which was a sum of the number of locations where pain was reported (range: 0-6). Participants were also asked: “in the past month, how much has this pain or discomfort restricted your daily activities” and “in the past month, how much has this pain and discomfort kept you from getting a good night's sleep” with “a lot,” “some,” or “not at all” as response options. Participants who responded “a lot” or “some” were considered to have pain that limited their daily activities or pain that interfered with their sleep, respectively. Lastly, participants were asked “have you ever seen a doctor about your pain” (yes/no) and “do you take any medication for your pain” (yes/no). These specific pain questions were assessed at Waves 7 through 9 of the H-EPESE, except for pain lasting longer than 4 weeks, which was assessed only at Waves 7 and 8.

#### Covariates

Sociodemographic and health characteristics were included as covariates in these analyses. These included age, sex (male/female), marital status (married, not married), education, nativity (US born, non-US born), language of interview (English, Spanish), MMSE score (36), and high depressive symptoms, measured with the Center for Epidemiologic Studies Depression Scale (CES-D ≥ 16) (37). Education status and nativity were measured at Wave 7, while age, sex, marital status, language of interview, MMSE score, and depressive symptoms were time-varying and measured at each wave.

### Statistical Analysis

Chi-Square Tests of Independence, Wilcoxon rank-sum tests, and *t*-tests were used to test the baseline descriptive characteristics of participants by multimorbidity status. We used general estimation equations to estimate the odds ratio (OR) and 95% Confidence Interval (CI) of (1) pain on weight-bearing; (2) pain location; and (3) pain that limits daily activities, over 6 years as a function of multimorbidity. We conducted a sensitivity analysis using general estimation equations to estimate pain location as a function of individual diseases included in our definition of multimorbidity. To understand how our findings may have been impacted by the exclusion of those with lower MMSE scores, we conducted a second sensitivity analysis where we examined the descriptive statistics of pain by cognitive impairment (MMSE < 21) (38, 39). For our third and fourth sensitivity analyses, we used general estimation equations to estimate the odds of pain location as a function of multimorbidity among those only with MMSE ≥ 21 and our full sample, with no inclusions, respectively. All analyses adjusted for sociodemographic and health characteristics described above. Sex, education status, and nativity were analyzed as time-stable covariates. Age, marital status, language of interview, MMSE score, depressive symptoms, pain, and comorbidities were measured at each wave and analyzed as time-varying, with the potential to change over time. Stata 17.0 was used for all analyses (StataCorp, LLC, College Station, TX).

## Results

### Sample Characteristics

Overall, our sample (*n* = 841) was 85.8 years old on average [standard deviation (SD): 3.9] and 64.1% were female (Table 1). Each participant could be interviewed one to three times, contributing up to 6 years of follow-up. On average, each participant completed 1.7 interviews and the average length of follow-up was 1.8 years. At baseline, 77.3% of participants had multimorbidity, while the remaining 22.7% did not. Overall, participants were more often unmarried, had 5.2 years of education on average, were majority born in the US, mostly completed interviews in Spanish, and had an average MMSE score of 21.2. However, those with multimorbidity were more often female and reported more depressive symptoms (*p* < 0.05). Those with multimorbidity had higher reported rates of all health conditions compared to those without multimorbidity. Hypertension, arthritis, and diabetes were the most common conditions reported among both groups. Among those with multimorbidity, the most common co-occurring conditions were hypertension and arthritis, hypertension and diabetes, arthritis and diabetes, arthritis and osteoporosis, and hypertension and heart failure.

**Table 1:** Baseline descriptive characteristics of Mexican Americans aged 80 and older by multimorbidity status (*n* = 841).

		**Multimorbidity**		
		***n*** **(%)**		
**Baseline characteristics**	**Total**	**Yes**	**No**	***P*** **-value**
	**(*n* = 841)**	**(*n* = 650; 77.3%)**	**(*n* = 191; 22.7%)**	
Age (years), mean (SD)	85.8 (3.9)	85.8 (3.8)	86.0 (4.2)	0.466
Gender^*^				<0.001
Male	302 (35.9%)	205 (31.5%)	97 (50.8%)	
Female	539 (64.1%)	445 (68.5%)	94 (49.2%)	
Marital Status				0.303
Married	265 (31.5%)	199 (30.6%)	66 (34.6%)	
Not married	576 (68.5%)	451 (69.4%)	125 (65.4%)	
Years of education	5.2 (4.1)	5.1 (4.1)	5.4 (4.0)	0.366
US Born	467 (55.5%)	354 (54.5%)	113 (59.2%)	0.250
English interview	148 (17.6%)	116 (17.9%)	32 (16.8%)	0.727
MMSE score	21.2 (6.9)	21.2 (6.8)	21.0 (6.9)	0.713
High depressive symptoms^*^	211 (25.1%)	182 (28.0%)	29 (15.2%)	<0.001
Average number of conditions (SD)^*^	2.7 (1.6)	3.3 (1.2)	0.65 (0.8)	<0.001
Diabetes^*^	294 (35.0%)	282 (43.4%)	12 (6.3%)	<0.001
Hypertension^*^	616 (73.3%)	559 (86.0%)	57 (29.8%)	<0.001
Arthritis^*^	540 (64.2%)	504 (77.5%)	36 (18.9%)	<0.001
Heart Attack^*^	73 (8.7%)	73 (11.2%)	0 (0.0%)	<0.001
Heart Failure^*^	225 (26.8%)	219 (33.7%)	6 (3.1%)	<0.001
Hip Fracture^*^	55 (6.5%)	52 (8.0%)	3 (1.6%)	0.002
Osteoporosis^*^	235 (27.9%)	229 (35.2%)	6 (3.1%)	<0.001
Liver Disease^*^	59 (7.0%)	58 (8.9%)	1 (0.5%)	<0.001
Kidney Disease^*^	91 (10.8%)	88 (13.5%)	3 (1.6%)	<0.001
COPD^*^	77 (9.2%)	76 (11.7%)	1 (0.5%)	<0.001
**Most common disease combinations**				
Hypertension + Arthritis		424 (50.4%)		
Hypertension + Diabetes		251 (29.9%)		
Arthritis + Diabetes		199 (23.7%)		
Arthritis + Osteoporosis		199 (23.7%)		
Hypertension + Heart Failure		189 (22.5%)		

* *p < 0.05 SD, standard deviation; MMSE, mini mental state examination; COPD, chronic obstructive pulmonary disease*.

**Figure 2:**
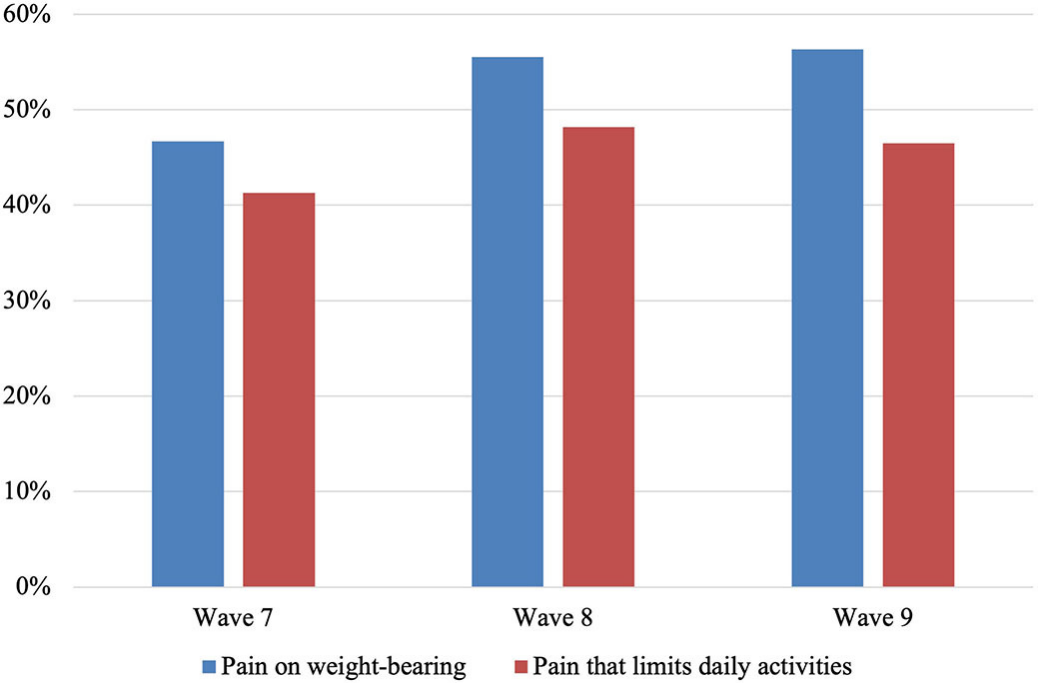
Percentage of Mexican Americans aged 80 and older reporting pain on weight-bearing and pain that limits daily activities (a lot or somewhat) at each wave of interview (*n* = 841).

Figure 2 displays the percentage of participants reporting pain on weight-bearing and pain that limits daily activities (a lot or somewhat) at each wave. At Wave 7 (baseline), 46.7% of participants reported pain on weight-bearing; this increased to 55.5% at Wave 8 and 56.3% at Wave 9. At Wave 7, 41.3% of participants reported pain that limited their activities a lot or somewhat, which increased to 48.2% at Wave 8, then slightly decreased to 46.5% at Wave 9.

**Figure 3:**
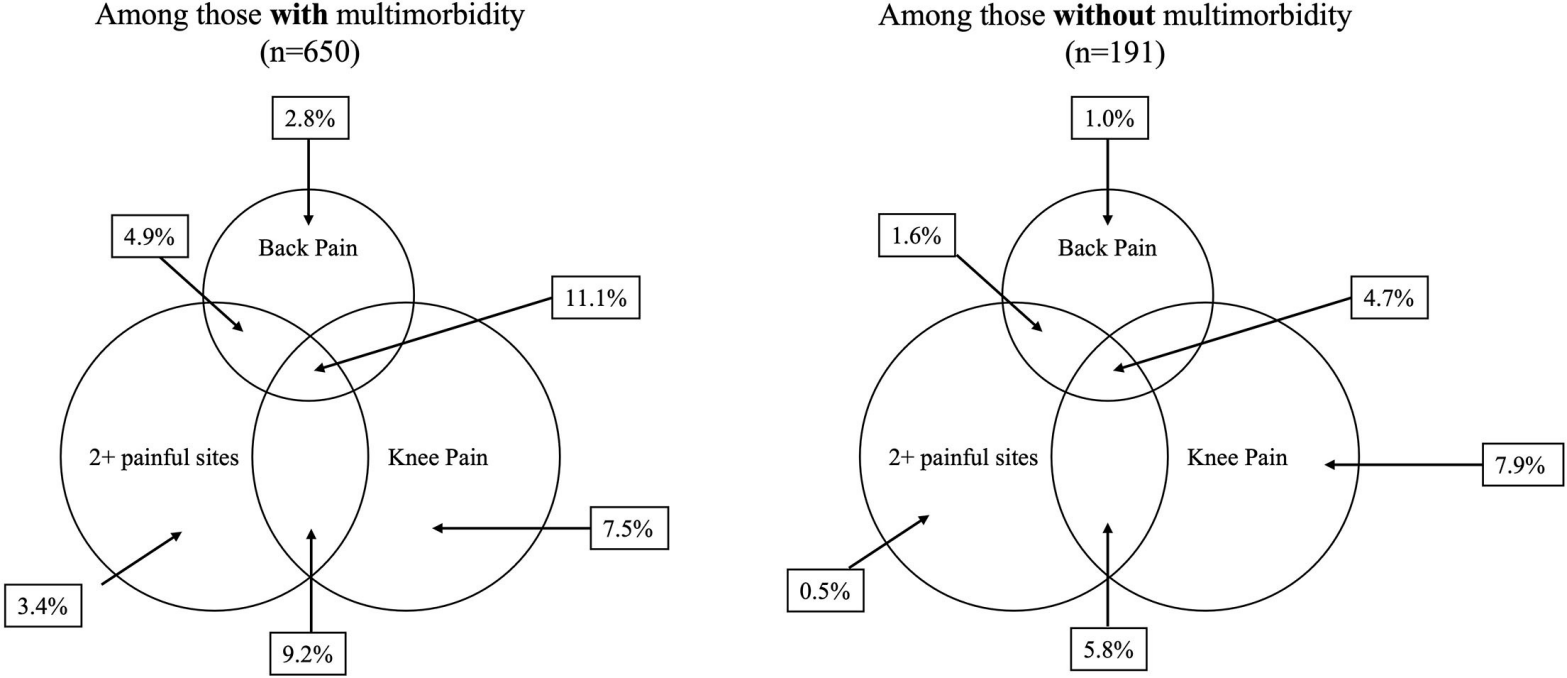
Overlapping pain locations by multimorbidity status at baseline among Mexican Americans aged 80 (*n* = 841).

Participants with multimorbidity more often reported pain on weight-bearing (*p* < 0.05) (Table 2). The most commonly reported pain locations were knees, legs, and ankles/feet for those both with and without multimorbidity; however, those with multimorbidity more frequently reported each pain location. The median number of pain locations was 0.0 for both those with and without multimorbidity; however, those with multimorbidity had a wider interquartile range (0.0-2.0) compared to those without multimorbidity (0.0-1.0) (*p* < 0.05). Among those with multimorbidity, 28.6% reported pain at two or more locations, compared to 12.6% of those without multimorbidity (*p* < 0.05). Those with multimorbidity more frequently reported pain that limited their daily activities a lot (19.8% vs. 6.8%) or somewhat (25.7% vs. 19.9%) (*p* < 0.05). Among those reporting pain, there was no difference in pain lasting longer than 4 weeks, pain interfering with sleep, reporting doctor visits for pain, or taking medication for pain, based on multimorbidity status. Overall, most participants reported pain lasting longer than 4 weeks (83.0%), seeing a doctor for pain (89.3%), and taking medication for pain (79.1%). Figure 3 displays the overlapping pain locations by multimorbidity status. Among those with multimorbidity, 11.1% reported pain at two or more sites, including back and knee pain, compared to 4.7% of those without multimorbidity. Additionally, 9.2% of participants with multimorbidity reported pain at two or more sites, including knee pain but not back pain, compared to 5.8% of those without multimorbidity.

**Table 2:** Baseline pain experiences of Mexican Americans aged 80 and older by multimorbidity status (*n* = 841).

		**Multimorbidity**		
**Total**		***n*** **(%)**		
**Baseline pain variables**	**Total**	**Yes**	**No**	***P*** **-value**
	**(*n* = 841)**	**(*n* = 650; 77.3%)**	**(*n* = 191; 22.7%)**	
Pain on weight-bearing^*^				<0.001
Yes	393 (46.7%)	336 (51.7%)	57 (29.8%)	
No	448 (53.3%)	314 (48.3%)	134 (70.2%)	
**Pain in:**				
Back^*^	136 (16.2%)	122 (18.8%)	14 (7.3%)	<0.001
Knees^*^	216 (25.7%)	181 (27.9%)	35 (18.3%)	0.008
Ankles/feet^*^	153 (18.2%)	132 (20.3%)	21 (11.0%)	0.003
Legs^*^	198 (23.5%)	173 (26.6%)	25 (13.1%)	<0.001
Hips^*^	113 (13.4%)	105 (16.2%)	8 (4.2%)	<0.001
Entire body^*^	66 (7.9%)	60 (9.2%)	6 (3.1%)	0.006
2 or more locations^*^	210 (25.0%)	186 (28.6%)	24 (12.6%)	<0.001
Median number of pain locations (IQR)^*^	0.0 (0.0-1.0)	0.0 (0.0-1.0)	0.0 (0.0-2.0)	<0.001
Pain limits daily activities^*^				<0.001
A lot	142 (16.9%)	129 (19.8%)	13 (6.8%)	
Somewhat	205 (24.4%)	167 (25.7%)	38 (19.9%)	
No	494 (58.7%)	354 (54.5%)	140 (73.3%)	
**Among those with pain (*n* = 393)**				
Pain lasted > 4 weeks	326 (83.0%)	281 (83.6%)	45 (79.0%)	0.385
Pain interfered with sleep				0.373
A lot	58 (14.8%)	53 (15.8%)	5 (8.8%)	
Somewhat	149 (37.9%)	125 (37.2%)	24 (42.1%)	
Not at all	186 (47.3%)	158 (47.0%)	28 (49.1%)	
Ever seen doctor for pain?				0.070
Yes	351 (89.3%)	304 (90.5%)	47 (82.5%)	
Take medication for pain?				0.092
Yes	311 (79.1%)	272 (81.0%)	39 (68.4%)	

* *p < 0.05 IQR, interquartile range*.

### Fully Adjusted General Estimating Equations Models

**Figure 4:**
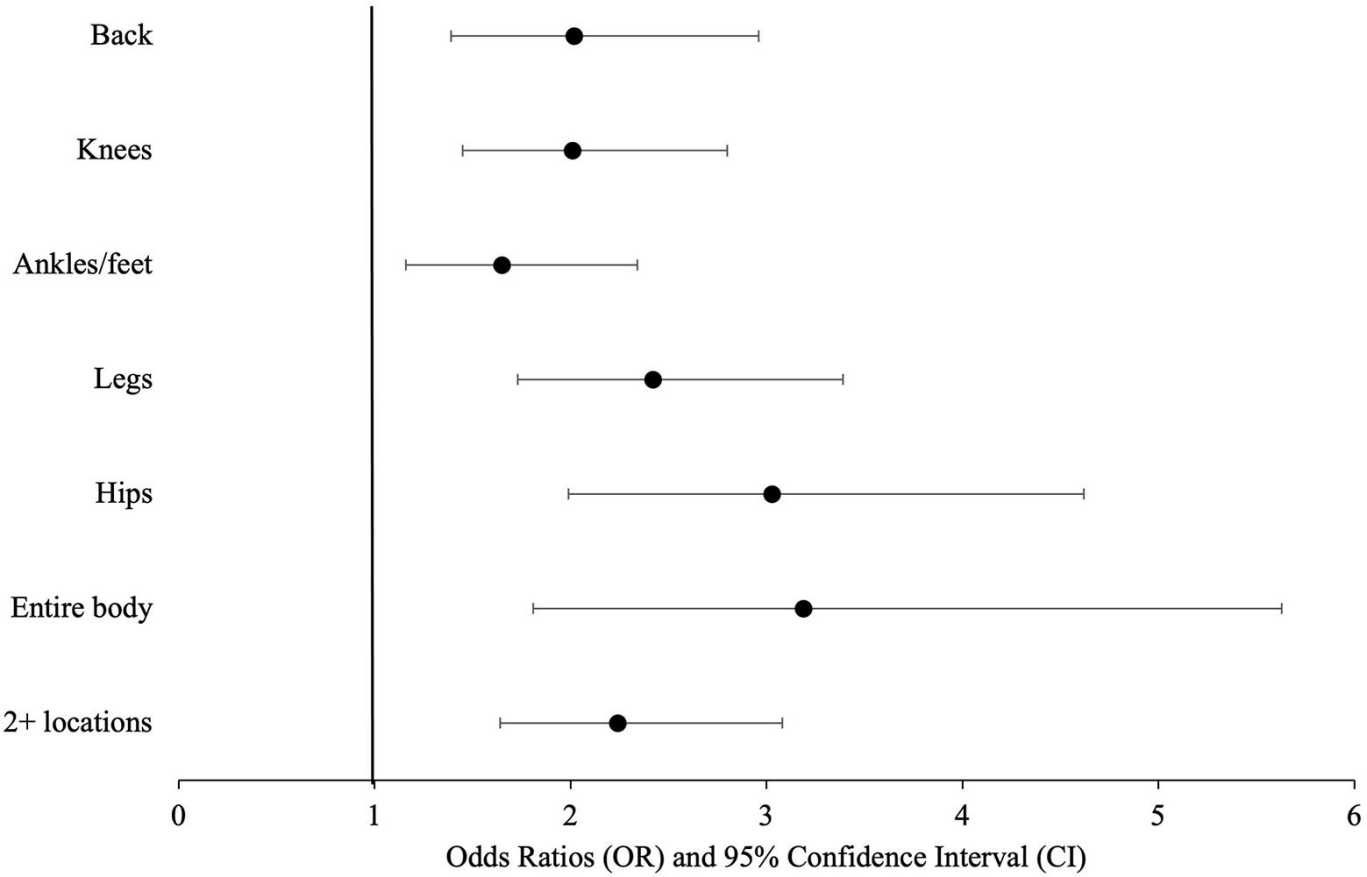
Odds ratios and 95% confidence intervals of pain location as a function of multimorbidity among Mexican Americans aged 80 and older (*n* = 841).

Those with multimorbidity had greater odds (OR = 2.27, 95% CI: 1.74, 2.95) of reporting pain on weight-bearing and pain that limited their daily activities (OR = 2.12, 95% CI: 1.61, 2.78) over time, compared to those without multimorbidity (Table 3). Those with high depressive symptoms had higher odds of pain on weight-bearing and pain that limits daily activities, compared to those with low depressive symptoms. Higher MMSE scores were associated with lower odds of pain that limits daily activities. Those with multimorbidity had higher odds of reporting pain at each pain location and of reporting pain at two or more locations compared to those without multimorbidity (Figure 4).

**Table 3:** Fully adjusted general estimating equation models for pain as a function of multimorbidity status over 6 years among Mexican Americans aged 80 and older (*n* = 841).

**Participant characteristics**	**Pain on weight-bearing** **OR (95% CI)**	**Pain that limits daily activities** **OR (95% CI)**
Multimorbidity	**2.27 (1.74, 2.95)**	**2.12 (1.61, 2.78)**
Age	1.00 (0.97, 1.03)	1.00 (0.97, 1.03)
Female	0.94 (0.72, 1.22)	1.11 (0.85, 1.46)
Years of education	0.99 (0.96, 1.02)	0.98 (0.95, 1.02)
Married	1.01 (0.78, 1.31)	1.15 (0.88, 1.49)
US Born	0.95 (0.74, 1.23)	1.01 (0.78, 1.30)
Spanish interview	1.12 (0.83, 1.53)	1.32 (0.95, 1.85)
MMSE score	0.99 (0.97, 1.00)	**0.98 (0.96, 0.99)**
High depressive symptoms	**1.69 (1.35, 2.12)**	**1.88 (1.50, 2.35)**

*OR, odds ratio; CI, confidence interval; MMSE, mini mental state examination. The bold values indicate p < 0.05*.

We conducted a sensitivity analysis, where we included each disease individually (Supplementary Table 2). Those with arthritis had higher odds of reporting pain on weight-bearing and pain that limited daily activities compared to those without arthritis or high depressive symptoms. Those who reported osteoporosis had higher odds of reporting pain on weight-bearing compared to those without osteoporosis. For our second sensitivity analysis, among our analytical sample (*n* = 841), 39.1% of participants had MMSE < 21 while 60.9% had an MMSE ≥ 21. We found that, among those with MMSE < 21, 51.1% reported pain on weight-bearing while 44.0% of those with MMSE ≥ 21 reported pain on weight-bearing (*p* = 0.043). In our third sensitivity analysis, among only those with MMSE ≥ 21 in our analytical sample (*n* = 512), we found consistent results where multimorbidity was associated with pain on weight-bearing (OR: 2.12; 95% CI: 1.53, 2.94) and pain that limits daily activities (OR: 1.90; 95% CI: 1.36, 2.65). The results of our fourth sensitivity analysis, where we included all H-EPESE participants (*n* = 1,078), regardless of complete baseline data, were fairly consistent but more conservative for pain on weight-bearing (OR: 2.04; 95% CI: 1.61, 2.58) and pain that limits daily activities (OR: 1.91; 95% CI: 1.50, 2.44).

## Discussion

In our study of community-dwelling Mexican Americans aged 80 and over, we examined the association of multimorbidity with pain, pain location, and pain that limited daily activities over 6 years of follow up. We found that about 77% of participants had multimorbidity; of these, over half (51.7%) reported pain on weight-bearing. Multimorbidity was associated with higher odds of pain on weight-bearing, pain that limited their daily activities in the past month, pain at each pain location, and overlapping pain at two or more locations.

Our findings are consistent with previous work that found a link between pain and multimorbidity (21, 23–25, 27). For example, a study of multimorbidity and pain among adults aged 18 and older in Canada found that both pain and multimorbidity were common, and the odds of reporting pain increased with both multimorbidity and the number of multimorbid conditions (25). This latter finding was also observed in New Zealand among adults aged 15 and older, where those with multimorbidity had higher odds of reporting chronic pain (21). Other studies, defining multimorbidity as the presence of three or more chronic diseases, conducted among those aged 65 and older in Germany (23) and those 75 and older in Sweden (24), have documented a high prevalence of pain in those with multimorbidity.

The results presented in this study build on the literature on the association between multimorbidity and pain, as few studies on multimorbidity have examined locations of pain, how pain interferes with daily activities, and overlapping pain sites. We found that, among those with multimorbidity, almost 30% reported pain at two locations, compared to about 13% of those without multimorbidity. When we examined overlapping knee, back, and multisite pain by multimorbidity status, we found that those with multimorbidity reported knee, back, and multisite pain over twice as often as those without multimorbidity. This result is consistent with previous work using data from the UK data bank among participants aged 37-73 who documented a dose-response relationship between number of long-term chronic conditions and pain (27).

The intersection of pain and multimorbidity has clinical implications. Most evidence-based guidelines have limited applicability to individuals with co-morbid chronic conditions (23, 40). Because pain and multimorbidity are common among older adults, it is important for providers to determine which patient co-morbidities may impact physical function (22). While interventions are often targeted at one chronic pain condition (e.g., back pain) (16), older adults with multimorbidity may require multimodal interventions to optimally control pain that may be present at two or more body sites (e.g., overlap of back and knee pain) or various types of pain (e.g., nociceptive vs. neuropathic), with each site or type requiring a different approach to achieve improvement in function and quality of life (41). Managing chronic pain in individuals with multiple chronic conditions represents a challenge, as the adverse effects of pain medication, such as opioids or non-steroidal anti-inflammatory drugs, may be amplified by the presence of other chronic diseases, such as cardiovascular disease or chronic kidney disease (42). Polypharmacy may represent a challenge among older adults with both pain and multimorbidity (43).

The mechanisms underlying pain and multimorbidity are not well understood. However, a direct potential mechanism may be dysregulation of physiological mechanisms and accumulated disruptions across multiple systems, which is associated with increasing age and the presence of multimorbid diseases and frailty syndrome (44, 45). Other potential mechanisms include the stress caused by adaptation to living with and management of chronic disease (46). On the other hand, pain and multimorbidity share many risk factors; identifying distinct risk factors is a crucial step toward diagnosis, prevention, and management of these two conditions (22). It is important for future work to consider the presence of chronic overlapping pain conditions (16, 22).

### Limitations

Our findings should be considered along with some limitations. First, selection bias may have occurred through the inclusion of individuals aged 80 and older at baseline. This may have resulted in a more robust, healthier sample, and may have underestimated the true relationship between multimorbidity and pain. Second, our findings may be susceptible to recall bias due to our older population and self-reported health conditions. Third, we may have underestimated the relationship between multimorbidity and pain, as those who were excluded had lower MMSE scores and more often reported pain. Fourth, since our sample was restricted to Mexican Americans living in the Southwestern US, our findings may be not generalizable to the Hispanic population in the US. The Hispanic population is heterogeneous and pain burden differs by country of origin, as older adults from different nationalities may have different health profiles and life course experiences that influence their development of pain (31, 47). Despite these limitations, our study strengths include the examination of site and functional impact of pain and multimorbidity longitudinally (6 years), the inclusion of comprehensive socioeconomic and health factors, the location of pain and pain interference with daily activities, overlapping pain locations (knee, back, and multisite pain), and the use of the H-EPESE survey, a well-categorized sample of very old Mexican Americans.

### Conclusions

In this sample of very old community-dwelling Mexican Americans, those with multimorbidity consistently had higher odds of pain, including pain at two or more locations, highlighting the need for early management of pain among those with multiple chronic conditions and complex health needs. This is especially important among very old Mexican Americans, who have a high burden of chronic health conditions. Very old Mexican Americans are a growing, yet understudied, group, and studies on pain and multimorbidity are mostly based on non-Hispanic White cohorts. Future work is needed to understand the mechanisms underlying overlapping pain conditions and multimorbidity, especially in the context of the syndemic and intersectionality frameworks (16–18).

## Data Availability Statement

Publicly available datasets were analyzed in this study. This data can be found at: National Archive of Computerized Data on Aging [https://www.icpsr.umich.edu/web/NACDA/series/546].

## Ethics Statement

The studies involving human participants were reviewed and approved by Institutional Review Board at the University of Texas Medical Branch (IRB # 92-85). The patients/participants provided their written or oral consent to participate in this study.

## Author Contributions

SAM was responsible for study design, analyses, interpretation of the results, and writing of the manuscript. SAS was involved in study design, analyses, interpretation of the results, and editing of the manuscript. YFK, DSL, KSM, and MAR were responsible for study design, interpretation of the results, and editing of the manuscript. All authors contributed to the article and approved the submitted version.

## Funding

SAM was supported by a Research Career Development Award (K12HD052023: Building Interdisciplinary Research Careers in Women's Health Program-BIRCWH; Berenson, PI) from the National Institutes of Health/Office of the Director (OD)/National Institute of Allergy and Infectious Diseases (NIAID), and Eunice Kennedy Shriver National Institute of Child Health and Human Development (NICHD). The Hispanic Established Populations for Epidemiological Studies of the Elderly (H-EPESE) was funded by the National Institutes of Health, National Institute on Aging (R01AG10939; Markides, PI). YFK and MAR were supported by the National Institute on Drug Abuse (R01-DA039192; YFK, MAR, MPI). SA was supported by the National Institute on Aging (R01 MD010355; SA—PI). DSL was supported by the National Institutes of Health (NIH) and National Institute on Aging, Grant # P30 AG059301; and Cancer Prevention and Research Institute of Texas, Grant # RP210130. This work was also supported by the Texas Resource Center on Minority Aging Research (P30AG059301, Markides, PI) and the Claude D. Pepper Older Americans Independence Center (P30AG024832, Volpi, PI).

## Author Disclaimer

The content is solely the responsibility of the authors and does not necessarily represent the official views of the National Institutes of Health.

## Conflict of Interest

The authors declare that the research was conducted in the absence of any commercial or financial relationships that could be construed as a potential conflict of interest.

## Publisher's Note

All claims expressed in this article are solely those of the authors and do not necessarily represent those of their affiliated organizations, or those of the publisher, the editors and the reviewers. Any product that may be evaluated in this article, or claim that may be made by its manufacturer, is not guaranteed or endorsed by the publisher.
