# Supplementary material for: Multimorbidity Is Associated With Pain Over 6 Years Among Community-Dwelling Mexican Americans Aged 80 and Older
Source: Front Pain Res (Lausanne). 2022 Mar 23;3:830308. doi: 10.3389/fpain.2022.830308 (PMC8983931; doi:10.3389/fpain.2022.830308)
Supplement: Supplementary file 1 [file Data_Sheet_1.pdf]

Supplementary Table 1: Baseline descriptive characteristics of the sample by included vs. excluded participants.

	Included		
**Baseline Characteristics**	**Yes (n=841; 78.0%)**	**No (n=237; 22.0%)**	**p-value**
**Age (years), mean (SD)**	85.8 (3.9)	86.3 (4.2)	0.095
**Gender**			0.181
Male	302 (35.9%)	74 (31.2%)	
Female	539 (64.1%)	163 (68.8%)	
**Marital Status**			0.896
Married	265 (31.5%)	73 (31.1%)	
Not married	576 (68.5%)	162 (68.9%)	
**Years of education***	5.2 (4.1)	4.4 (3.9)	0.008
**US Born**	467 (55.5%)	121 (51.1%)	0.222
**English Interview**	148 (17.6%)	48 (20.3%)	0.349
**MMSE Score (SD)***	21.2 (6.9)	17.5 (9.6)	<0.001
**High Depressive Symptoms***	211 (25.1%)	54 (36.7%)	0.003
**Multimorbidity**	650 (77.3%)	190 (80.2%)	0.345
**Pain on Weight-Bearing**	393 (46.7%)	134 (82.7%)	<0.001
**Pain that Limits Daily Activities**	347 (41.3%)	99 (60.7%)	<0.001

*p<0.05

Note: SD=standard deviation; MMSE=Mini Mental State Examination.

Supplementary Table 2: Fully adjusted general estimating equation models for pain as a function of individual diseases over 6 years among Mexican Americans aged 80 and older reporting pain on weight-bearing (n=841).

Participant Characteristics	Pain on Weight-Bearing<br>OR (95% CI)	Pain that Limits Daily Activities<br>OR (95% CI)
**Diabetes**	0.94 (0.73, 1.20)	0.94 (0.73, 1.20)
**Hypertension**	1.33 (1.00, 1.75)	1.27 (0.94, 1.72)
**Arthritis**	**2.99 (2.33, 3.82)**	**2.79 (2.16, 3.61)**
**Heart Attack**	0.91 (0.61, 1.35)	0.80 (0.54, 1.17)
**Heart Failure**	1.04 (0.81, 1.35)	1.16 (0.90, 1.51)
**Hip Fracture**	0.66 (0.42, 1.04)	0.67 (0.44, 1.03)
**Osteoporosis**	**1.36 (1.02, 1.81)**	1.33 (0.99, 1.77)
**Liver Disease**	0.97 (0.60, 1.58)	0.81 (0.52, 1.27)
**Kidney Disease**	1.17 (0.84, 1.62)	1.28 (0.92, 1.78)
**Age**	0.99 (0.96, 1.03)	1.00 (0.97, 1.03)
**Female**	0.82 (0.62, 1.09)	1.00 (0.75, 1.35)
**Years of Education**	1.00 (0.97, 1.03)	0.99 (0.96, 1.03)
**Married**	0.99 (0.76, 1.30)	1.13 (0.86, 1.48)
**US Born**	0.97 (0.75, 1.26)	1.02 (0.78, 1.33)
**Spanish Interview**	1.11 (0.82, 1.52)	1.32 (0.94, 1.86)
**MMSE Score**	0.98 (0.97, 1.00)	**0.97 (0.96, 0.99)**
**High Depressive Symptoms**	**1.64 (1.29, 2.09)**	**1.83 (1.44, 2.32)**

Note: COPD was not included due to small cell sizes. OR=odds ratio; CI=confidence interval; MMSE=Mini Mental State Examination. Bold values indicate $p<0.05$
